# Perceptions of COVID-19 Vaccine Incentives Among Adolescents and Young Adults

**DOI:** 10.1001/jamanetworkopen.2022.16628

**Published:** 2022-06-08

**Authors:** Caroline M. Hogan, Marika E. Waselewski, Parker Szachta, Clara Wolff, Xochitl Amaro, Tammy Chang

**Affiliations:** 1 Susan B. Meister Child Health Evaluation and Research Center, National Clinician Scholars Program, Institute for Healthcare Policy and Innovation, Department of Pediatrics, University of Michigan, Ann Arbor; 2 Department of Family Medicine, University of Michigan, Ann Arbor; 3 University of Michigan, Ann Arbor; 4 Institute for Healthcare Policy and Innovation, Department of Family Medicine, University of Michigan, Ann Arbor

## Abstract

**Importance:**

Vaccine incentives have been used across the US to encourage COVID-19 vaccine uptake and include programs targeted to adolescents and young adults. However, little is known about youths’ views regarding these initiatives.

**Objective:**

To assess experiences and perceptions of COVID-19 vaccine incentives in a nationwide sample of US youth.

**Design, Setting, and Participants:**

A qualitative survey study was conducted using the MyVoice text message-based polling platform from October 22 to October 29, 2021. Participants were US adolescents and young adults aged 14 to 24 years, who were sent 5 open-ended questions to assess their experiences and perceptions of COVID-19 vaccine incentives. Qualitative responses were analyzed thematically. Descriptive statistical analysis was performed in January 2022.

**Main Outcomes and Measures:**

Experiences, perceptions, and opinions related to COVID-19 vaccine incentives as measured by survey response.

**Results:**

A total of 1125 of 1206 youth (93%) responded to the survey and had a mean (SD) age of 20 (2) years, 664 (59%) identified as male, and 769 (68%) identified as non-Hispanic White. Of respondents, 871 (79%) reported having heard of vaccine incentives, and 892 (82%) believed they were a good idea or had positive attributes. Notably, 305 youth (28%) expressed concerns about vaccine incentives, citing uncertainty about their effectiveness (86 [28%]), their ethical use (63 [21%]), the impact on vaccine motivations (51 [17%]), and confidence (39 [13%]), and lack of fairness (35 [11%]). Only 73 youth (7%) reported that an incentive influenced their decision to get a COVID-19 vaccine. When asked what they thought would motivate others to get a COVID-19 vaccine, youth with an opinion (536 of 1032 [52%]) most commonly reported incentives (112 [21%]) and additional COVID-19 vaccine testing, safety, or regulation (115 [21%]).

**Conclusions and Relevance:**

COVID-19 vaccine incentives are well known to youth but not a significant self-reported motivator for vaccination. Although generally viewed favorably, more than a quarter of youth expressed concerns regarding vaccine incentives, including but not limited to their ethics, effectiveness, fairness, and impacts on vaccine motivation and confidence. Policymakers considering targeted use of COVID-19 vaccine incentives should weigh youths’ perspectives on these initiatives alongside objective effectiveness and cost-effectiveness data.

## Key Points

**Question:**

What do US adolescents and young adults know and think about COVID-19 vaccine incentives?

**Findings:**

In this qualitative study of 1125 adolescent and young adult respondents, youth awareness of COVID-19 vaccine incentives was high, and their opinions were generally favorable. However, more than a quarter of youth expressed concerns about incentives, including but not limited to their effectiveness, ethical use, fairness, and impact on vaccine motivations and confidence.

**Meaning:**

These findings suggest that more research is needed to understand the incidence, characteristics, and effectiveness of COVID-19 vaccine incentives targeted to children and young adults. Policymakers considering interim implementation of incentive programs should consider youths’ perspectives on these public health measures.

## Teaser

This qualitative study assesses experiences and perceptions of COVID-19 vaccine incentives in a nationwide sample of 1125 adolescents and young adults in the US.

## Introduction

Throughout 2021, US state and local governments, public health organizations, insurers, and private businesses introduced incentives to encourage COVID-19 vaccine uptake. Some of these programs were targeted to adolescents and young adults, who make up approximately 14% of the population and thus represent a key demographic in the country’s COVID-19 vaccination campaign.^1,2^ Lotteries, scholarships, cash payments, event tickets, and free items are just some of the incentives that have been offered to vaccinated adolescents, college students, and other young adults.^3,4^

While there has not yet been a comprehensive characterization of youth COVID-19 vaccine incentives, news reports suggest they are relatively common and can be significant in scope. For example, California offered children 12 years of age and older who received at least 1 dose of a COVID-19 vaccine the opportunity to enter a lottery with $1.5 million prizes,^5^ and, in Minnesota, fully vaccinated 12 to 17-year-olds could win $100 000 college scholarships.^6^

Despite the implementation of COVID-19 vaccine incentives, little is known about youths’ views regarding these initiatives. Understanding youth perspectives is important given the significant financial resources made available for COVID-19 vaccine incentives^7^; a lack of consensus around incentive effectiveness^8,9,10,11,12,13,14^; and the ethical considerations of offering incentives to promote vaccine uptake,^15,16,17^ particularly to minors less than 18 years of age who, in most states, can neither independently consent to vaccination nor participation in COVID-19 incentive programs. This qualitative survey study aims to identify adolescent and young adults’ experiences and perceptions of COVID-19 vaccine incentives, with the goal of shaping future public health campaigns and investments targeted to these groups.

## Methods

This qualitative study was approved by the University of Michigan institutional review board with a waiver of parental consent for minors given the minimal risk to participants. All participants provided written consent during online enrollment. This study followed American Association for Public Opinion Research (AAPOR) reporting guideline for survey research^18,19^ and the Standards for Reporting Qualitative Research (SRQR) reporting guideline.^20^

The survey was fielded using MyVoice, a national text message-based polling platform of US youth. Participants ranged from age 14 to 24 years and were recruited from social media based on national benchmarks from the American Community Survey.^21^ Demographic information, including self-reported age, gender, race and ethnicity, education level, parental education level, free or reduced lunch status, and region were collected at study enrollment.^22^ Participants received a small payment of US $1 for responding to the survey.

Data on race and ethnicity were collected to ensure the MyVoice youth cohort mirrors, as much as possible, weighted demographic characteristics from the American Community Survey. Participants were asked the question, "What is your race? Check all that apply." They were given the following response categories to choose from: “American Indian or Alaska Native, Asian, Black or African American, Native Hawaiian or other Pacific Islander, White or Caucasian, and other (please describe).” To collect ethnicity data, participants were also asked, "Are you Hispanic or Latino?" with a yes or no response option.

Five open-ended questions focused on COVID-19 vaccine incentives were sent to 1206 youths on October 22, 2021. These questions were developed and analyzed by a team of researchers with clinical experience in pediatrics and adolescent medicine and methodologic expertise in qualitative mixed methods research. The questions included: (1) Have you heard about incentives for getting the COVID-19 vaccine (lotteries, scholarships, free stuff, etc)? If yes, what have you heard of? (2) Do you think incentives are a good idea? Why or why not? (3) Did an incentive influence your decision about getting vaccinated? Tell us about it. (4) If you have not gotten vaccinated against COVID-19, what would it take for you to get vaccinated? (5) If someone you know has not gotten vaccinated, what would it take for them to get vaccinated? The survey was closed to additional responses on October 29, 2021.

### Data Analysis

Qualitative responses were analyzed thematically using a grounded theory approach. Four investigators (C.M.H., M.E.W., P.S., C.W.) iteratively identified response categories for the 5 questions and created a shared codebook based on these themes. Each question was then coded by a pair of investigators (C.M.H., M.E.W., P.S., C.W., or X.A.), who independently analyzed all responses to that question. Any discrepancies in coding were discussed until consensus was reached. Frequency statistics were calculated using Excel 2016 (Microsoft) in January 2022.

## Results

Among the 1125 participants who responded to at least 1 question (1125 of 1206; 93% response rate), the mean (SD) age was 20 (2) years, 664 (59%) identified as male, 769 (68%) identified as non-Hispanic White, and 462 (41%) qualified for free or reduced lunch (Table 1). We did not directly ask participants if they were vaccinated against COVID-19; however, 832 of 1043 (80%) of respondents reported receiving a COVID-19 vaccine in their free text responses. Representative quotes from respondents for each question can be found below and in Table 2, along with notable code frequencies.

**Table 1.** Demographic Characteristics of Survey Respondents and Nonrespondents From the MyVoice Cohort

Characteristics	No. (%)		ACS 2019, weighted %
	Respondents (n = 1125)	Nonrespondents (n = 81)	
Age, mean (SD)	20 (2)	19 (2)	NA
Gender			
Male	664 (59)	37 (46)	51.2
Female	360 (32)	36 (44)	48.8
Other	101 (9)	8 (10)	NA
Race and ethnicity			
Hispanic	100 (9)	20 (25)	23.4
Non-Hispanic			
Black	72 (6)	14 (17)	13.8
White	769 (68)	32 (40)	52.5
Other^a^	182 (16)	15 (19)	10.3
Education level			
Less than high school^b^	256 (23)	26 (33)	42.4
High school graduate	141 (13)	13 (16)	21.7
Some college or technical school	483 (43)	28 (35)	24.6
College or technical degree	245 (22)	13 (16)	11.3
Region			
Midwest	320 (28)	25 (31)	21.0
Northeast	252 (22)	9 (11)	16.6
South	319 (28)	31 (39)	38.5
West	232 (21)	15 (19)	23.9
Free or reduced lunch recipient			
Yes	462 (41)	41 (52)	NA
No	657 (59)	38 (48)	NA

Abbreviations: ACS, American Community Survey; NA, not applicable.

^a^ The other category includes respondents who identified as American Indian or Alaska Native, Asian, Native Hawaiian or other Pacific Islander, or self-identified as "other," with a free-text option to describe.

^b^ Includes participants still in high school.

**Table 2.** Questions and Representative Respondent Quotes for the Most Common Themes

Theme	No. (%)^a^	Representative quote(s)
**Have you heard about incentives for getting the COVID-19 vaccine (lotteries, scholarships, free stuff, etc)? If yes, what have you heard of? (n = 1106)**		
Yes	871 (79)	“Yes”; “I have”
Lotteries or raffles	361 (41)	“Lotteries”; “Sweepstakes mainly”
Money or cash-equivalent	275 (32)	“I’ve heard that they’re giving $100-1000 to people who get the vaccine.”
Free items	211 (24)	“Apple products”; “marijuana cigarettes”; “discounts…at online stores or local stores in my city”
Food and drink	163 (19)	“Krispy Kreme’s free donut everyday for the rest of the year”
Scholarships	158 (18)	“University scholarships”; “free tuition and housing”
Event tickets/experiences	58 (7)	“Free lollapalooza tickets”
		“Tickets to sports games”
Services	30 (3)	“Yes free uber rides”
No/unsure	235 (21)	“No, I honestly haven’t heard of those kinds of incentives.”
**Do you think incentives are a good idea? Why or why not? (n = 1092)**		
Yes	759 (70)	“Yea, it may motivate people who were otherwise hesitant but needed a push”
No	172 (16)	“No it seems sketchy”
Yes and no	133 (12)	“Yes, but I think it can also send the wrong message”
Unsure/maybe	28 (3)	“Perhaps I’m not sure”
Supporting reasons		
Effective or cost-effective	459 (51)	“Yes because it encourages more people to get the vaccine”
		“...You pay a little to get more vaccinated and you save a lot in care costs for patients with covid-19”
Supports common good	91 (10)	“Yes because it protects everyone from spreading the virus”
Fun reward	89 (10)	“Yes, they try to get people to take the vaccine in a more fun way”
Needed for normalcy	67 (8)	“Yes, because as many people as possible need to be vaccinated in order to get through this.”
Money motivates	59 (7)	“Yeah, I think they are because it motivated people who are on the fence or are reward motivated”
Opposing reasons		
Not effective or cost-effective	86 (28)	“No because ppl don’t want to get the shot”
		“No. They waste money”
Unethical	63 (21)	“No, it is bribing people to get a vaccine that they may not want”
Wrong motivations	51 (17)	“Incentives can make people feel entitled to rewards.”
Promotes mistrust	39 (13)	“They aren’t, I don’t trust them.”
Unfair	35 (11)	“I think they are unfair to the millions of people who got vaccinated without being eligible for an incentive”
**Did an incentive influence your decision about getting vaccinated? Tell us about it. (n = 1081)**		
Yes	73 (7)	“Yes I did it for the incentive”
Lotteries or raffles	11 (15)	“Yes, thinking that I could be the winner of $1 million encouraged me to get vaccinated faster.”
Money or cash-equivalent	10 (14)	“Yes. I was short on cash, so it really helped”
Food and drink	9 (12)	“Yes, I received a free food order for 1 month at a supermarket chain”
Free items	8 (11)	“Really yes because I had already decided not to get vaccinated but they gave discounts in the sports store to those vaccinated and I got vaccinated”
No	1008 (93)	“No, I did not consider receiving any of those incentives to get vaccinated.”
Unaware or unavailable	167 (17)	“No, there are no incentives in my state”
		“No, I got vaccinated before most incentives started rolling out”
Motivated by safety or illness concerns	202 (20)	“The only incentive I needed was having a better chance at staying healthy and alive.”
		“No, I got vaccinated to protect those around me.”
Motivated by desire for normalcy	21 (2)	“No, I thought it was a blessing enough to be able to get the vaccine and get back to being normal afterwards”
Motivated by mandates/requirements	20 (2)	“No, but my employer at the time was starting to mandate it”
Motivated by desire to socialize	18 (2)	“Yeah, i got one so I could be able to see my friends and go out”
Motivated by parents or family	15 (1)	“No my mother had made me get the vaccine”
**If you have not gotten vaccinated against COVID-19, what would it take for you to get vaccinated? (n = 1043)**		
Already vaccinated	832 (80)	“I am vaccinated because I feel like it is my duty as a member of a society, I don’t want to be a part of spreading this disease”
Nothing (strong refusal)	19 (2)	“You could not get me vaccinated, it’s not possible.”
Unsure	16 (2)	“I don’t know honestly”
Specific motivators	177 (17)	
Incentive	56 (32)	“Free money would’ve driven me to get vaccinated along with free college tuition for four years”
Vaccine research, safety, and/or approval	38 (21)	“At least a year or so of testing to see if there are any long term side effects”
		“Probably FDA authorization”
COVID-19 impact (self or other)	33 (19)	“A very strong reason like someone getting sick ”
Mandates/requirements	15 (8)	“Stuff like having to be fully vaccinated to eat in a restaurant or go certain places”
Access	14 (8)	“It needs to be easy to get, location and time”
**If someone you know has not gotten vaccinated, what would it take for them to get vaccinated? (n = 1032)**		
Family/friends/peers already vaccinated	164 (16)	“Everyone I know has been vaccinated at this point”
Nothing (strong refusal)	108 (10)	“Nothing at this point, people aren’t gonna do it if they haven’t”
Unsure	224 (22)	“I have no idea about that.”
Specific motivators	536 (52)	
Vaccine research, safety, and/or approval	115 (21)	“More proof that the vaccine is safe, like full authorization or authorization for more age groups”
Incentive	112 (21)	“Probably a money incentive. That friend…wasn’t vaccinated until that $50 gift card. Then they got vaccinated.”
Information or education	69 (13)	“Explaining the science behind it and why it’s safe and make the community safer”
COVID-19 impact (self or other)	64 (12)	“They would get vaccinated if they had a risk to their safety”
Mandates/requirements	58 (11)	“Requirement by employer or government”
Change in mindset	55 (10)	“They must have confidence to go out to get vaccinated”
External support or pressure	37 (7)	“It would take me convincing them, because they would be able to see how important it is”

^a^ Totals may not add to 100% as codes are not mutually exclusive.

### Awareness of COVID-19 Vaccine Incentives and Concern About Unintended Consequences

When asked, 871 youth (79%) reported having heard of COVID-19 vaccine incentives. The most well-known incentive types among this group included lotteries and raffles (361 [41%]), cash payments and cash equivalents (275 [32%]), free items (ranging from “stickers” and “free donut[s]” to “firearms” and “weed”) (211 [24%]), food and drink (163 [19%]), scholarships (158 [18%]), and event tickets and other experiential incentives (such as “free vacations”) (58 [7%]). Less frequently identified incentive types included free or discounted services (such as “manicure[s]” and “free rides from Uber”) (30 [3%]) and getting time off from work or school (8 [1%]).

Most respondents (892 [82%]) thought that vaccine incentives were a good idea or had positive attributes, citing beliefs that incentives were effective or cost-effective in promoting vaccine uptake (459 [51%]), beneficial for the “greater good” of society (91 [10%]), or a fun or otherwise “nice reward” for getting vaccinated (89 [10%]). There were also beliefs that vaccine incentives were necessary and would facilitate a “return to normal” or prepandemic existence (67 [8%]). A few respondents reported feeling that incentives were a preferable alternative to vaccine mandates (19 [2%]).

A total of 305 respondents (28%) had concerns about COVID-19 vaccine incentives; of these, most worried that incentives were ineffective and/or cost-ineffective tools to promote vaccination (86 [28%]), unethical or akin to “bribery” (63 [21%]), created “wrong reason[s]” or motivations to get vaccinated (51 [17%]), decreased trust in vaccines and the institutions promoting vaccination (39 [13%]), or were unfair (particularly for “the millions of people who got vaccinated without being eligible for an incentive”) (35 [11%]). Thirty respondents (3%) expressed frustration that incentives were even necessary, with a respondent stating, “[we] shouldn’t have to bribe people to not die.”

### Participants’ Experiences With Incentives

Only 73 respondents (7%) reported that an incentive influenced their decision to get a COVID-19 vaccine. Of those who were motivated by an incentive, the most commonly reported incentive types were lotteries and raffles (11 [15%]), cash payments and gift cards (10 [14%]), food and drink (9 [12%]), free items (8 [11%]), event tickets and other experiential incentives (5 [7%]), scholarships (4 [5%]), free or discounted services (2 [3%]), and getting time off from work or school (2 [3%]).

More youth reported not being motivated by incentives (1008 [93%]) and expressed a nonincentive-related reason for getting vaccinated. These included to stay healthy and otherwise not “get or spread COVID” (202 [20%]), to “get back to normal,” and return to a prepandemic existence (21 [2%]), to comply with a vaccine mandate (20 [2%]), to visit friends or family, or otherwise engage in social activities (18 [2%]), or because a family member asked them to or “made [them]” (15 [1%]). Of note, when asked what they thought would motivate others to get a COVID-19 vaccine, incentives were one of the most common responses for youth with an opinion (112 [21%]).

### Opinions About Other Methods of Increasing COVID-19 Vaccination

When asked what it would take for an unvaccinated acquaintance to get a COVID-19 vaccine, 332 respondents (32%) reported being unsure (224 [22%]) or that “nothing” could convince these individuals (108 [10%]). Additionally, 164 respondents (16%) reported that all their acquaintances were already vaccinated. Of the 536 respondents with an opinion about how to promote vaccine uptake, the most commonly reported factors were additional COVID-19 vaccine testing or safety data (115 [21%]), incentives (112 [21%]), more general education or information-sharing about vaccines (69 [13%]), getting COVID-19, or having “someone close to them [get it]” (64 [12%]), and vaccine mandates (58 [11%]).

Fewer respondents felt that the following interventions would be effective: social support or pressure to get vaccinated (37 [7%]); improved access to vaccination (28 [5%]); a recommendation from a doctor, religious leader, politician, or other respected source (27 [5%]); more trust in government, science, and/or health care institutions (18 [3%]); permission from a family member (7 [1%]); or an alternative vaccine formulation, such as “a nasal mist option” (7 [1%]).

## Discussion

Our study reported that most youth have been exposed to a variety of incentives to promote COVID-19 vaccine uptake, ranging from lotteries and cash payments to item giveaways and scholarships. Despite youths’ widespread exposure to vaccine incentives, most of our respondents denied that their personal decision to get a COVID-19 vaccine was influenced by an incentive. More common was a desire to stay healthy or minimize the spread of COVID-19 to respondents’ friends, family members, and the general public. However, when asked what they thought would motivate others to get a COVID-19 vaccine, about 20% of youth with an opinion reported incentives, suggesting a possible lack of youth insight into their true vaccination motivations or a disconnect between the perceived vs actual effectiveness of vaccine incentives in this demographic.

It is possible that the right type of incentive program could motivate youth vaccination. For example, a pilot program that provided $25 cash cards to North Carolinians who received or drove someone to receive their first COVID-19 vaccine dose effectively slowed a regional decline in vaccination.^13^ Adolescents and young adults, whose social connections are foundational to their identity and development, may be more responsive to these types of social incentives than to others.

Although most respondents believed that vaccine incentives were a good idea, 28% expressed concerns about incentives. Beyond skepticism about the effectiveness of these programs, these youth had more fundamental concerns about the ethics of vaccine incentives and expressed worry that incentives undermined altruistic vaccine motivations, contributed to vaccine mistrust, and decreased vaccine confidence, and were unfair to those who were vaccinated without an incentive. Some ethicists have raised similar concerns about COVID-19 vaccine incentives in recent analyses^15^; this study suggests that those concerns are shared by a segment of US youth.

As of May 2022, about one-sixth of the eligible US population has not received any COVID-19 vaccine.^23^ More than half of the respondents in our study offered specific strategies to promote vaccine uptake among the unvaccinated. These included generating more vaccine-specific testing, safety data, or regulation; providing more education and information about vaccines in general; and implementing vaccine mandates. Some respondents felt that improved vaccine access, recommendations from trusted leaders, or developing noninjection vaccine formulations would be beneficial. About 6% of respondents felt that the only way to increase vaccination was through personal experience, with the unvaccinated either *“*getting sick” themselves or having “someone close to them… get very sick or die.”

Almost all COVID-19 vaccine incentive research to-date has been focused on the adult population; this survey study is the first, that we know of, to elicit youth opinions on vaccine incentives. More research is needed to evaluate the scope and characteristics of youth COVID-19 vaccine incentives and to evaluate the effectiveness of these programs in promoting vaccine uptake. Policymakers and investigators should continue to weigh the ethical considerations of offering vaccine incentives to children who, in most states, cannot independently consent to vaccination and require their parent or guardian’s consent to enroll in vaccine incentive programs.

### Limitations

This study had limitations. While MyVoice recruits youth based on age, gender, race and ethnicity, and geographical benchmarks for national data, respondents are not nationally representative because there is no assurance that recruitment methods will reach all eligible participants. Certain groups of respondents (male gender, non-Hispanic White ethnicity and race, at least some college education, and Midwestern and Northeastern location) were overrepresented, which limits generalizability. Additionally, social media recruitment necessarily limits participation to those who use it, which also limits the generalizability of our results.

Another limitation stems from the vaccination status of our survey participants. While we purposefully did not ask participants if they were vaccinated against COVID-19, based on question responses, at least 80% of respondents received a COVID-19 vaccine. It is possible that unvaccinated respondents have substantively different opinions about incentives compared with their vaccinated counterparts. Relatedly, since our study provides monetary incentives for participation, it is possible the cohort is biased toward those who are motivated by incentives, however small.

## Conclusions

In this qualitative study of US adolescents and young adults, COVID-19 vaccine incentives are well-known but not a significant self-reported motivator for youth vaccination; however, they are perceived to be motivating to others. Although generally viewed favorably, 28% of youth respondents expressed concerns about vaccine incentives, including but not limited to their ethical use, effectiveness, and impact on vaccine motivation and confidence. More research is needed to better characterize COVID-19 vaccine incentives targeted to youth and to evaluate the effectiveness of these programs in promoting vaccine uptake. Policymakers and investigators should consider youths’ perspectives on COVID-19 vaccine incentives, along with the ethical implications of offering incentives to children who cannot independently consent to vaccination or participation in incentive programs.
